# Supplementary material for: Evidence-based action plan for integrating artificial intelligence in an academic medical centre-a multidisciplinary approach
Source: PLoS One. 2026 Apr 17;21(4):e0346582. doi: 10.1371/journal.pone.0346582 (PMC13089692; doi:10.1371/journal.pone.0346582)
Supplement: Attachment — Submitted filename: October 2025 point by cpoint response.docx [file pone.0346582.s001.docx]

October 2025

To the Editor-in-Chief

PLOS ONE

**Subject: Rebuttal letter for article titled- “Evidence-Based Action Plan for Integrating Artificial Intelligence in an Academic Medical Centre-A Multidisciplinary Approach”**

Dear Sir,

Thank you for your feedback and guidance. We have addressed all concerns raised point by point below.

- Thank you for submitting your manuscript to PLOS ONE. After careful consideration, we feel that it has merit but does not fully meet PLOS ONE’s publication criteria as it currently stands. Therefore, we invite you to submit a revised version of the manuscript that addresses the points raised during the review process. To progress toward peer review, the manuscript must be revised to include a detailed and explicit discussion of study's limitations, potential biases, and the justification or calculation for the sample size.

Response: Study now includes limitations, potential biases, and the justification or calculation for the sample size.

- Furthermore, to comply with the journal's reporting standards, the COREQ checklist must be fully completed and submitted alongside the revised manuscript. Once these changes are made and the required checklist is provided, we will assess the manuscript's suitability for progression to the peer-review process.

Response: the COREQ checklist has been submitted now.

- When submitting your revision, we need you to address these additional requirements. Please ensure that your manuscript meets PLOS ONE's style requirements, including those for file naming. The PLOS ONE style templates can be found at <https://journals.plos.org/plosone/s/file?id=wjVg/PLOSOne_formatting_sample_main_body.pdf> and <https://journals.plos.org/plosone/s/file?id=ba62/PLOSOne_formatting_sample_title_authors_affiliations.pdf>

Response: We have revised the manuscript according to the PLOS ONE style requirements and ensured that all files are renamed and formatted in line with the provided templates including the title page.

- We note that your Data Availability Statement is currently as follows: “All relevant data are within the manuscript and its Supporting Information files.” Please confirm at this time whether or not your submission contains all raw data required to replicate the results of your study. Authors must share the “minimal data set” for their submission. PLOS defines the minimal data set to consist of the data required to replicate all study findings reported in the article, as well as related metadata and methods (<https://journals.plos.org/plosone/s/data-availability#loc-minimal-data-set-definition>). For example, authors should submit the following data:- The values behind the means, standard deviations and other measures reported;- The values used to build graphs;- The points extracted from images for analysis. Authors do not need to submit their entire data set if only a portion of the data was used in the reported study. If your submission does not contain these data, please either upload them as Supporting Information files or deposit them to a stable, public repository and provide us with the relevant URLs, DOIs, or accession numbers. For a list of recommended repositories, please see <https://journals.plos.org/plosone/s/recommended-repositories>. If there are ethical or legal restrictions on sharing a de-identified data set, please explain them in detail (e.g., data contain potentially sensitive information, data are owned by a third-party organization, etc.) and who has imposed them (e.g., an ethics committee). Please also provide contact information for a data access committee, ethics committee, or other institutional body to which data requests may be sent. If data are owned by a third party, please indicate how others may request data access.

Response: We have already shared the themes, even codes and analysis of the qualitative data. The full data cannot be shared due to confidentiality restrictions. However all relevant data supporting the findings including coding, themes etc are described in the paper. The study can be replicated using the details provided in the manuscript.

- If the reviewer comments include a recommendation to cite specific previously published works, please review and evaluate these publications to determine whether they are relevant and should be cited. There is no requirement to cite these works unless the editor has indicated otherwise.

Response: Not applicable

Regards,

Corresponding Author:

**Dr Lena Jafri**

Visiting Faculty

Chemical Pathology

Department of Pathology and Laboratory Medicine,

The Aga Khan University, Pakistan

Phone: 92-213-4861927

Email: [lena.jafri@aku.edu](mailto:lena.jafri@aku.edu)
