# Supplementary material for: Evidence-based action plan for integrating artificial intelligence in an academic medical centre-a multidisciplinary approach
Source: PLoS One. 2026 Apr 17;21(4):e0346582. doi: 10.1371/journal.pone.0346582 (PMC13089692; doi:10.1371/journal.pone.0346582)
Supplement: Attachment — Submitted filename: PONE-D-25-46411R1.docx [file pone.0346582.s002.docx]

**Major revision:**

1. Clarity in methodology:
   - The methods Phase I mentions “grounded theory”, but the described procedures are closer to qualitative descriptive or thematic/content analysis.

Either:

- Strengthen the claims of grounded theory elements (e.g., explicit constant comparison, theoretical sampling, analytic memos, movement toward a substantive theory),

OR

- Rewrite the approach as qualitative descriptive / thematic analysis and remove claims of grounded theory.
- Make the analytic approach explicit and consistent across Phases I and II (e.g., “conventional content analysis” or “reflexive thematic analysis”) and describe it once clearly, then reference back to it.

2. Strengthen sampling description and justification

- Provide a clear, concise description of Inclusion criteria (who qualified as “academic leaders/clinicians”) and any exclusion criteria.
- How many potential participants were invited, how many declined, and reasons if known.
- How data saturation was assessed (e.g., “no new codes emerged after interview X; two additional interviews were conducted to confirm”).

3. The critical scientific contribution is how interview and workshop data were translated into the SWITCH-structured action plan. Currently this is described narratively but not analytically. Describe how and which of the phase 1 and 2 outcomes were mapped to SWITCH model.
4. At which stage SWITCH model introduced? Before phase 1 and 2 or after? And elaborate on the basis of inclusion.

**Minor revisions:**

- Some typographical and grammatical errors in the manuscript need correction (e.g., “particpants”, “Hoewever”, “saturtation”, “regualtions”, inconsistent spacing)
- Expand the abbreviations when used first time.
- Abstract can be improved methodology with sampling and analysis
