# Supplementary material for: Evidence-based action plan for integrating artificial intelligence in an academic medical centre-a multidisciplinary approach
Source: PLoS One. 2026 Apr 17;21(4):e0346582. doi: 10.1371/journal.pone.0346582 (PMC13089692; doi:10.1371/journal.pone.0346582)
Supplement: Attachment — Submitted filename: Jan 2026 response to plos one reviewers SWITCH.docx [file pone.0346582.s003.docx]

Jan 2026

To

The Editor-in-Chief

PLOS ONE

**Subject: Response to reviewers’ comments for article titled “Evidence-Based Action Plan for Integrating Artificial Intelligence in an Academic Medical Centre-A Multidisciplinary Approach”**

Dear Sir,

We have revised the manuscript in accordance with the academic editor’s and reviewers’ suggestions. A point-by-point response is provided below, along with both a tracked-changes version and a clean revised manuscript submitted on the PLOS One portal.

1. Specifically, the authors should revise the abstract to provide a concise scientific summary that includes a clear justification for the chosen methods, a brief description of the analytic procedures, and concrete, validated outcomes. Additionally, restructure the introduction to clearly articulate a specific research gap and validate the selection of the SWITCH model over alternative frameworks.

Response: Abstract has been revised as per suggestions. We clarify that the aim of this study was exploratory and developmental, focusing on the co-creation of a practical framework rather than the validation of outcomes. We have revised the manuscript to clarify the exploratory nature of the study, explicitly describe the analytic process, articulating a clearer research gap, and reason behind choosing SWITCH over other models of change for devising the action plan.

2. In the methods section, adopt a coherent qualitative design by clearly defining whether you are using a grounded-theory approach or alternate method. Provide a justification for the prospective sample size and address aspects of researcher reflexivity, along with triangulation through explicit procedures.

Response: We agree with reviewers. In this study, the grounded theory approach has been removed in favor of an exploratory research design that used thematic analysis. This method aimed to uncover unknown patterns and themes from interview and workshop discussion data, employing an inductive approach to ensure findings were data-driven and allowed themes to emerge organically from participants' responses, aligning with the exploratory nature of the study. By employing the concepts of information power and theme saturation to clearly justify the anticipated sample size, we have now improved the Methods section. Additionally, we have included a clear reflexivity statement and described the triangulation techniques used, such as data source and analyst triangulation.

3. Finally, deepen the results and action plan sections by incorporating richer, analytically driven themes, including illustrative quotations and a validated, prioritized implementation framework. The discussion must also critically reflect on methodological limitations and ethical considerations around AI usage, power dynamics, structural constraints.

Response: Results have been edited, added more quotations, tried writing it analytically with interpretations where needed. Since this was beyond the scope of the study, the action plan was not validated; however, the SWITCH model of change framework was employed, and the results were integrated into the SWITCH framework. The Discussion has been revised focusing on literature review, ethics and limitations of the study.

4. Reviewer #1: Abstract-The abstract is largely descriptive and reads more like a project summary than a scientific abstract. Critical elements are missing, including a clear justification for methodological choices, explicit analytic procedures, and concrete outcomes beyond thematic labels. Claims of providing a “structured strategic action plan” are not supported by evidence of validation, implementation, or evaluation.
   Response: The abstract has been revised as per these suggestions of writing it scientifically. We wish to clarify that the manuscript does not claim formal validation or implementation of the action plan. Rather, it presents a conceptual, structured action plan developed from qualitative findings and aligned with the SWITCH model, intended to guide institutions in AI integration. We have revised the text to make this distinction clearer and avoid any potential misunderstanding.
5. Introduction-The introduction provides a generic overview of AI in healthcare and change management theories but lacks a sharply articulated research gap. The rationale for selecting the SWITCH model over other established frameworks remains underdeveloped and largely asserted rather than justified. The novelty of the study is unclear, particularly given the abundance of conceptual papers on AI integration and leadership in healthcare.

Response: The Introduction has been strengthened to clearly articulate the existing gaps, novelty and the rationale for conducting this study. The SWITCH model was selected for its focus on practical behaviour change among healthcare stakeholders, rather than just organizational restructuring. It uniquely incorporates cognitive, emotional, and environmental factors, suitable for the study's multi-phase design involving interviews, discussions, and workshops over six months. This flexibility allowed its application across various study stages and stakeholder groups. The manuscript has been revised to more clearly justify the selection of the SWITCH model (in the introduction).

6. Methods-This is the weakest section of the manuscript. The study is labeled as “qualitative cross-sectional,” yet the design blends interviews, workshops, and consensus meetings without a coherent qualitative methodological framework.

Response: Thank you for this comment, we have revised the methods section and agree and the study design has been modified too.

7. Methods-Claims of using grounded theory are not supported by methodological rigor (e.g., no constant comparison, no theory generation, no memoing).

Response: In this study, the grounded theory approach has been removed in favor of an exploratory research design that used thematic analysis. This method aimed to uncover unknown patterns and themes from interview and workshop discussion data, employing an inductive approach to ensure findings were data-driven and allowed themes to emerge organically from participants' responses, aligning with the exploratory nature of the study. We have revised the Methods section to remove references to grounded theory, as the study did not aim to generate a formal theory.

8. Methods-Sample size justification is post-hoc and conceptually weak; reliance on a pilot of two interviews to justify adequacy is not defensible.

Response: We respectfully clarify that the sample size was not determined post hoc. The proposed sample size of interviews was specified a priori and approved by the institutional ethics review committee before data collection commenced. We agree, however, that our original explanation relied too heavily on the pilot interviews. We have therefore revised the Methods section to remove the pilot based justification and now explicitly ground the sample size rationale in established qualitative principles, including purposive sampling of information-rich participants and anticipated thematic saturation.

9. Methods-The role of researchers (many of whom are institutional insiders and co-authors) raises significant concerns regarding reflexivity, positionality, and social desirability bias, which are insufficiently addressed. Triangulation is asserted but not demonstrated analytically.

Response: Agree, this has been addressed in the methods section now. We have updated the Methods section to specifically consider researcher positionality, including prior experience and institutional familiarity. Confidentiality was guaranteed to participants, and open-ended questions were used in neutral, non-evaluative interviews to promote candid observations. Throughout the process of gathering + analyzing data, researchers kept written reflections to track how their viewpoints might have affected interpretations. To increase credibility and lower the possibility of social desirability bias, results were also triangulated across participant views and cross-checked with pertinent documents whenever feasible.

10. Results-The results are largely descriptive and repetitive.

Response: This was a helpful comment. We agree that earlier version of results were too descriptive. Results section has been revised by the authors and we have tried to remove repetition and tried to strengthen analytical depth.

11. Results-Themes are broad, unsurprising, and closely aligned with existing literature, offering limited analytical depth.

Response: We recognize that multiple themes presented align with existing literature. The themes were developed inductively and later discussed with workshop and meeting participants. In response to the reviewer's critique, we have enhanced the Results section by providing analytic commentary within each theme, elucidating the interconnections among themes to enhance interpretive depth.

12. Results-Coding appears largely confirmatory, reinforcing pre-existing assumptions about AI rather than generating new insights.

Response: We thank the reviewer for this comment. We respectfully clarify that the analysis was not intended to be confirmatory. Any alignment with existing literature would therefore have been unintentional. An inductive coding approach was employed, deriving codes from participants’ accounts instead of predefined theories. Acknowledging the initial presentation's shortcomings, the Methods and Results sections were revised to better articulate the inductive coding process and enhance the analytic commentary, emphasizing how participant perspectives influenced and expanded existing understandings of AI implementation.

13. Results-Quotations are sparse and selectively illustrative rather than analytically rich.

Response: We have revised the Results section as per this helpful suggestion to ensure that quotations are used analytically rather than illustratively. Pre or post quotation analysis have been added where appropriate. More quotes have been added. In the initial version quotations were used selectively to avoid overly descriptive results. But now we understand how relevant quotations can be added.

14. Results-Tables function more as checklists than as vehicles for theory building or interpretive synthesis.

Response: We agree with the reviewer and this is an important observation. We have tried to edit the tables as much as we could to strengthen this aspect of the paper.

15. Action Plan (Phase III)-The action plan is extensive but essentially prescriptive and aspirational. It resembles a policy or institutional guideline rather than a research output.

Response: We appreciate the reviewer’s recognition of the action plan’s scope and practical relevance. The action plan was analytically derived which is not coming through. We have clarified its analytic intent to ensure it is read as a research output rather than a prescriptive guideline.

16. Action Plan (Phase III)-No prioritization framework, feasibility testing, stakeholder validation beyond authors, or implementation metrics are provided. The plan is not empirically evaluated, piloted, or compared against alternatives, limiting its scholarly contribution.

Response: We appreciate the reviewer’s insightful critique and agree that empirical evaluation, feasibility testing, stakeholder validation and comparative analysis would strengthen the work. We wish to clarify that the primary aim of this manuscript is to propose a conceptual and integrative framework, rather than to report empirical findings. Please note, data collection (both in interviews and also in workshop) included many organizational leaders and decision-makers, who function as key stakeholders in the context of this study. This informally does provide stakeholder validation beyond the authorship team.

17. Discussion-The discussion reiterates results without critically engaging with methodological limitations or alternative interpretations. Claims of scalability and transferability are speculative and not supported by comparative data. The discussion conflates relevance with rigor; importance of topic does not compensate for methodological weakness. Engagement with critical AI ethics, power dynamics, and structural constraints in LMIC contexts remains superficial.

Response: Discussion has been rewritten taking account of literature, limitations and avoiding repetition of results. Moving beyond a summary of results, we now critically evaluate our findings against existing literature. While the importance of the topic remains a driver, we have reinforced the study's rigor by being more transparent about our methodological choices and the validity of our conclusions. Please note the findings from the current study offer potential insights into how healthcare institutes may integrate AI based on study findings. These insights are exploratory and provide a foundation for future empirical and comparative studies. Therefore, claims about scalability and transferability are presented as potential insights rather than confirmed outcomes. Further studies with larger samples and comparative data are needed to validate these aspects.

18. Limitation-Although a limitations section is present, it is largely formulaic and understated. Key issues such as insider bias, lack of independent validation, and absence of outcome assessment are not adequately acknowledged. The implications of these limitations for the credibility of the action plan are not discussed.

Response: Limitations section has been written and we have tried to address the potential biases, validations and outcome assessment need.

19. Reviewer #2: The paper has a relevant and timely focus on AI inclusion in medical education and research, and action plan using the SWITCH model), which could be attractive if methodological rigor, clarity, and positioning are strengthened. The core issues now are precision and depth in the qualitative methodology, sharper articulation of novelty, and substantial tightening of language and structure. These will enhance the article and will benefit wider audience.

Response: Thank you for the comments. As per reviewers’ and editor’s suggestions methods and results have been modified bringing clarity, analytical interpretation and relevance. We have tried to improve writing and structure of the manuscript too.
