# Supplementary material for: Evidence-based action plan for integrating artificial intelligence in an academic medical centre-a multidisciplinary approach
Source: PLoS One. 2026 Apr 17;21(4):e0346582. doi: 10.1371/journal.pone.0346582 (PMC13089692; doi:10.1371/journal.pone.0346582)
Supplement: Attachment — Submitted filename: response to reviewers AI paper mar 2026.docx [file pone.0346582.s004.docx]

**2026/03/16**

**To the Editor PLOS One**

**Subject: Response to Reviewers’ Comments**

Dear Editor,
Thank you for sharing the reviewers’ comments and as per their suggestions we have revised our manuscript. All comments are responded below against each point and manuscript has been edited accordingly. We have submitted our revised manuscript in 3 separate files:

1. This document detailing the changes made
2. a copy of the manuscript with track-changes
3. a clean copy of the revised manuscript

- Reviewer #1: The manuscript addresses a timely and contextually important issue; however, before it can be considered for publication, several substantive concerns require further attention. In particular,1. The study should explicitly and consistently define its methodological orientation (e.g., exploratory qualitative study using reflexive thematic analysis) and ensure alignment between research questions, data collection methods (interviews, workshop, meetings), and analytic procedures. Response: We thank the reviewer for the opportunity to further clarify our methodological alignment. We have now explicitly labelled the study as an exploratory qualitative study using reflexive thematic analysis in the opening of the Methods section. Now the study objectives align with the research question, methodology and data analysis.
- 2. Provide a clearer step-by-step account of how codes were generated, how themes were refined, and how decisions were made during analysis. A visual analytic map or thematic development table would enhance transparency. Response: The methodology section has been revised to explicitly detail the systematic steps of thematic analysis, ensuring greater clarity and transparency in the research process. We have added a Visual Analytic Map (Figure 1) to the Methodology section to enhance transparency. This figure illustrates the iterative process of manual coding and clarifies how the workshop acted as a validation bridge between the initial inductive analysis and the final deductive mapping to the Switch Model.
- 3. Move beyond descriptive summaries of what participants said. For each theme, include interpretive commentary that explains why these patterns matter, how they relate to existing literature, and what tensions or contradictions emerged. Response: Agreed, in each theme we have added interpretive commentaries where possible.
- 4. Integrate quotations analytically rather than illustratively. Each quote should be followed by interpretation that demonstrates how it advances conceptual understanding rather than merely exemplifying a theme. Response: agree all the quotations are now integrated analytically rather than illustratively.
- 5. Given the insider status of several authors, include a more explicit reflexivity statement detailing positionality, power dynamics, and how potential bias was managed throughout data collection and analysis. Response: We acknowledge our insider status within the hospital. To manage potential bias, data collection was framed as collaborative problem solving rather than a top down evaluation, reducing perceived hierarchies. Trustworthiness was further ensured through methodological triangulation, comparing interview data with workshop findings, and guaranteeing anonymity to encourage candid participation and mitigate social desirability bias. We have tried to explain this in the methods section.
- 6. Specify how interview findings, workshop discussions, and planning meetings were compared, reconciled, and synthesized into the final action plan. The analytic integration process requires clearer articulation. Response: This has been modifed and rewritten in the methodology.
- 7. Provide a more critical comparison with alternative change-management frameworks, explaining precisely what conceptual gap the SWITCH model fills in this context. Response: Thank you for pointing this out. We have now added the critical comparison of models of change now in the introduction section.
- 8. Clearly distinguish the action plan from a policy guideline by articulating the theoretical insights derived from the data. Consider presenting a prioritization matrix or conceptual model that demonstrates analytic synthesis rather than listing components. Response: We agree that a distinction between a policy guideline and a research-derived action plan is vital. We have utilized the Switch framework (Rider, Elephant, and Path check table 2) not merely as a categorization tool, but as a conceptual model for analytic synthesis. This framework allows us to demonstrate how the qualitative themes translate into specific behavioural and environmental changes. The action plan is intentionally structured for practical utility to ensure knowledge translation which is a key goal of applied qualitative research. While it may resemble a policy guideline in its clarity, it is distinguished by its inductive origin. To emphasize this, we have added explicit links between the proposed actions and the emergent themes from the data. All edits are in track changes.
- 9. Explicitly acknowledge insider bias, absence of external validation, lack of pilot testing, and absence of outcome evaluation, and discuss how these limitations affect the credibility and applicability of the proposed framework. Response: We thank the reviewer for this critical observation. We agree that the use of a specific model and the current stage of the framework's development present significant limitations to its immediate applicability. We have revised the Limitations section to explicitly discuss how the insider bias, lack of pilot testing and how the SWITCH model may have introduced interpretive bias and how the absence of external validation and outcome evaluation necessitates caution when generalizing these findings.
- Reviewer #2: Major strengths • Timely, practice‑oriented topic in an LMIC • Focus on AI integration in a large academic medical centre in Pakistan addresses a real gap,as most AI‑change papers are conceptual or from HICs. • Multi‑source qualitative design• Three phases (leadership interviews, multidisciplinary workshop, then action‑plan development) create a coherent, developmental trajectory from perceptions → co‑design → framework. • Inclusion of leaders, faculty, and students increases the breadth of perspectives despite a single‑site limitation.• Explicit change‑management lens (SWITCH)• Clear mapping of findings to “Direct the Rider / Motivate the Elephant / Shape the Path” gives the action plan a structured and theoretically anchored form. Response: thank you for these positive encouraging comments.
- Recommended modifications• In Abstract and Methods, standardize to one primary descriptor, e.g.: “We conducted an exploratory qualitative study using reflexive thematic analysis of interviews and a co‑design workshop, followed by collaborative development of an action plan.”At the start of Methods, add a short “Study design and phases” paragraph that clearly lists:Phase I: Individual semi‑structured interviews with institutional leaders and faculty.Phase II: Co‑design workshop with multidisciplinary participants, using SWITCH as a sensitizing framework.Phase III: Collaborative development of a SWITCH‑aligned action plan based on Phases I–II. Response: Thank you for this suggestion. We have standardized the primary descriptor of the study design throughout the Abstract and Methods sections to ensure clarity and consistency. As requested, a "Study design and phases" subsection has been added to the beginning of the Methods section.
- • In the discussion, there are many repetitions about the SWITCH model's benefits; keep it once at the beginning. Response: Thankyou we have now edited the discussion and benefits of SWITCH have been merged together.
- • Break the limitations into bullet points or paragraphs. Response: Agree, we have now written the study limitations as bullet points for clarity.
- • Check the conflict of interest statement and correct. Response: Thank you for pointing out this error. The statement has been rewritten.
- • Check for spelling mistakes (pliot, emperical, effectiveness, particpants). Response: thank you we have now removed all grammatical errors and spelling mistakes.
